# From At-Risk Mental State to Psychosis: Demographic Characteristics and Clinical Corelates of Individuals Who Transitioned to Psychosis

**DOI:** 10.1192/bjo.2024.112

**Published:** 2024-08-01

**Authors:** Ameer Khoso, Jordan Bamford, Inti Qureshi, Imran Chaudhry, Nusrat Husain

**Affiliations:** 1 Pakistan Institute of Living and Learning, Karachi, Pakistan; 2 University of Manchester, Manchester, United Kingdom; 3 MerseyCare NHS Foundation Trust, Prescot, United Kingdom

## Abstract

**Aims:**

The at-risk mental state (ARMS) describes individuals at high risk of developing schizophrenia or psychosis. This study aimed at exploring the demographic characteristics of individuals who transitioned to psychosis from a large multicenter factorial design trial.

**Methods:**

This was a secondary analysis of large multicenter randomised controlled trial of minocycline and/or omega-3 fatty acids added to treatment as usual for at-risk mental states. Participants (n = 326) were randomised to minocycline, omega-3, combined minocycline and omega-3 or to double placebo for 6 months. The primary outcome was transition to psychosis at 12 months.

**Results:**

Forty-five (13.8%) participants transitioned to psychosis. The mean age of participants was 23.31 (5.31 SD) and 15.6% no formal education, 8.9% primary, 48.9% matriculation, 8.9% intermediate and 15.6% graduation and above. Majority 66% of participants were male and 71.1% single, 66.7% living in a joint family, 44.4% were employed, 24% students, 17.8% household/housewife and 3% unemployed. Interestingly 36.8% participants had a family history of psychosis, followed by 21.0% any unknown mental illnesses, 15.8% bipolar disorder, 15.8% depression, 5.3% anxiety and 5.3% intellectual disability. The mean total score for the Prodromal Questionnaire was 8.93, with a standard deviation of 1.67. The mean score on the Comprehensive Assessment for At Risk Mental State (CAARMS) unusual thoughts was 3.98 (SD = 0.84), Non-Bizarre Ideas 3.64 (SD = 0.77), Perceptual Abnormalities 3.76 (SD = 0.71) and disorganized speech 2.49 (SD = 1.12). Participants had mean Social and Occupational Functioning (SOFAS) score of 66.67 which suggests moderate difficulty in social, occupational, or school functioning (e.g., few friends, conflicts with peers or co-workers).

**Conclusion:**

Transition to psychosis appears to have different demographic and clinical correlates which may have the causal relationship to transition. The cross-comparative studies are warranted to understand differences and similarities between the groups.

